# Step-by-Step: Fusion-guided prostate biopsy in the diagnosis and surveillance of prostate cancer

**DOI:** 10.1590/S1677-5538.IBJU.2018.0886

**Published:** 2019-12-17

**Authors:** Nima Nassiri, Lauren Beeder, Azadeh Nazemi, Kian Asanad, John Um, Inderbir Gill, Masakatsu Oishi, Suzanne L. Palmer, Manju Aron, Osamu Ukimura, Andre Luis de Castro Abreu

**Affiliations:** 1 Institute of Urology, Keck School of Medicine, University of Southern California, Los Angeles, CA, USA;; 2 Department of Urology, Graduate School of Medical Science, Kyoto Prefectural University of Medicine, Kyoto, Japan;; 3 Department of Radiology, Keck School of Medicine, University of Southern California, Los Angeles, CA, USA;; 4 Department of Pathology, Keck School of Medicine, University of Southern California, Los Angeles, CA, USA

## Abstract

**Objective:**

To provide a step-by-step technique for fusion-guided biopsy for prostate cancer diagnosis and surveillance.

**Materials and Methods:**

All men with clinical indications for image-guided biopsy undergo 3-Tesla multiparametric magnetic resonance imaging (mpMRI) first. Lesions identified on mpMRI are graded using the Prostate Imaging-Reporting and Data System version 2.0 (PI-RADS v2) grading system. At the time of biopsy, real-time 3-dimensional (3D) transrectal ultrasound (TRUS) imaging is acquired and elastically fused with the mpMRI. Both targeted biopsies of MRI-derived suspicious lesions (PI-RADS 3-5) and systematic 12-core biopsies are performed. Patients without suspicious lesion on mpMRI undergo 3D TRUS-guided biopsy in standard templated fashion. In men placed on active surveillance (AS), prior positive sites are revisited using the trajectory and tracking functions of the fusion biopsy software.

**Results:**

The advantages of MRI/TRUS fusion biopsy for prostate cancer diagnosis and surveillance are numerous. The 3D model created by elastic fusion of real-time TRUS imaging to mpMRI provides excellent visualization of prostate anatomy. Suspicious lesions on mpMRI can be accurately targeted, increasing detection of clinically significant prostate cancer. Biopsy trajectory visualization provides spatial localization of the trajectory of the cores within the prostate. Systematic biopsies are also taken in addition to targeted cores to minimize the effect of the mpMRI-invisible lesion. Prior positive biopsy sites can be tracked in men on AS.

**Conclusions:**

Combined, the added benefits of prior lesion identification, adequate mapping of prostate anatomy and suspicious lesions, biopsy-trajectory visualization, tracking of prior positive biopsy sites, and combined targeted and systematic cores may offer the most effective method for prostate cancer diagnosis and surveillance.

## ARTICLE INFO

**Available at: http://www.intbrazjurol.com.br/video-section/20180886_Nassiri_et_al**

**Int Braz J Urol. 2019; 45 (Video #25): 1277-8**

